# *N*-(3-Fluoro­benzo­yl)-*N*′,*N*′′-bis­(4-methyl­phen­yl)phospho­ric triamide

**DOI:** 10.1107/S1600536811045314

**Published:** 2011-10-29

**Authors:** Mehrdad Pourayoubi, Samad Shoghpour, Giuseppe Bruno, Hadi Amiri Rudbari

**Affiliations:** a Department of Chemistry, Ferdowsi University of Mashhad, Mashhad 91779, Iran; b Dipartimento di Chimica Inorganica, Villaggio S. Agata, Salita Sperone 31, Università di Messina, 98166 Messina, Italy

## Abstract

In the title compound, C_21_H_21_FN_3_O_2_P, the NH and P(=O) groups of the C(=O)NHP(=O) fragment are in a *syn* arrangement with respect to each other, as are the two amide H atoms of the two CH_3_–4-C_6_H_4_–NH moieties. In the crystal, mol­ecules are linked through N—H⋯O(=P) and N—H⋯O(=C) hydrogen bonds, forming *R*_2_^2^(8) and *R*_2_^2^(12) rings, which are arranged in chains parallel to [010].

## Related literature

For hydrogen-bond patterns in phospho­ric triamides of the formula *R*C(O)NHP(O)[N*R* ^1^ *R* ^2^]_2_ and *R*C(O)NHP(O)[NH*R* ^1^]_2_, see: Toghraee *et al.* (2011 ▶). For different cyclic hydrogen-bond motifs, see: Pourayoubi *et al.* (2011 ▶).
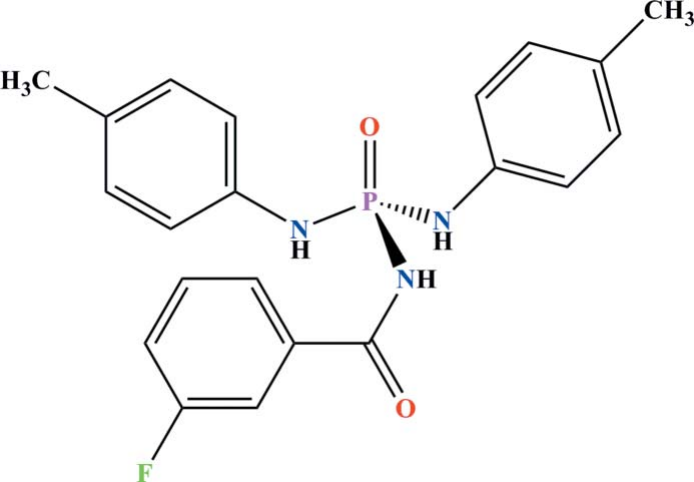


## Experimental

### 

#### Crystal data

C_21_H_21_FN_3_O_2_P
*M* *_r_* = 397.38
Monoclinic, 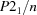

*a* = 10.2132 (5) Å
*b* = 9.8588 (4) Å
*c* = 20.2711 (9) Å
β = 93.621 (2)°
*V* = 2037.02 (16) Å^3^
*Z* = 4
Mo *K*α radiation
μ = 0.17 mm^−1^
*T* = 296 K
0.25 × 0.22 × 0.14 mm

#### Data collection

Bruker APEXII CCD diffractometer
Absorption correction: multi-scan (*SADABS*; Bruker, 2007 ▶) *T*_min_ = 0.662, *T*_max_ = 0.745
20105 measured reflections
3844 independent reflections
3061 reflections with *I* > 2σ(*I*)
*R*_int_ = 0.030

#### Refinement

*R*[*F* ^2^ > 2σ(*F* ^2^)] = 0.039
*wR*(*F* ^2^) = 0.109
*S* = 1.04
3844 reflections
255 parameters
H-atom parameters constrained
Δρ_max_ = 0.37 e Å^−3^
Δρ_min_ = −0.35 e Å^−3^

### 

Data collection: *APEX2* (Bruker, 2007 ▶); cell refinement: *SAINT* (Bruker, 2007 ▶); data reduction: *SAINT*; program(s) used to solve structure: *SHELXS97* (Sheldrick, 2008 ▶); program(s) used to refine structure: *SHELXL97* (Sheldrick, 2008 ▶); molecular graphics: *OLEX* (Dolomanov *et al.*, 2003 ▶); software used to prepare material for publication: *SHELXTL* (Sheldrick, 2008 ▶) and *enCIFer* (Allen *et al.*, 2004 ▶).

## Supplementary Material

Crystal structure: contains datablock(s) I, global. DOI: 10.1107/S1600536811045314/lh5349sup1.cif

Structure factors: contains datablock(s) I. DOI: 10.1107/S1600536811045314/lh5349Isup2.hkl

Additional supplementary materials:  crystallographic information; 3D view; checkCIF report

## Figures and Tables

**Table 1:** Hydrogen-bond geometry (Å, °)

*D*—H⋯*A*	*D*—H	H⋯*A*	*D*⋯*A*	*D*—H⋯*A*
N1—H1⋯O2^i^	0.86	1.97	2.7835 (18)	157
N3—H3⋯O1^ii^	0.86	2.06	2.8972 (18)	165

Symmetry codes: (i) 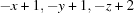; (ii) 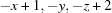.

**Fig. 1.**
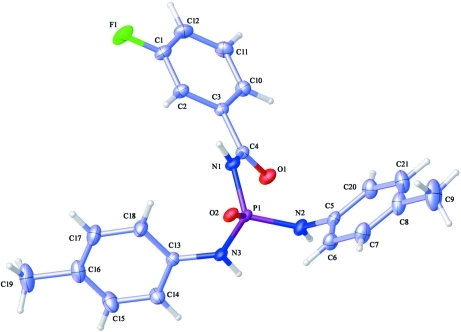
The molecular structure of the title compound. Ellipsoids are given at the 50% probability level.

## supplementary crystallographic information

### Comment

The possible hydrogen bond patterns in crystal structure of phosphoric triamides of the general formula *R*C(O)NHP(O)[N*R*^1^*R*^2^]_2_ and *R*C(O)NHP(O)[NH*R*^1^]_2_ have been analyzed recently (Toghraee *et al.*, 2011) and the hydrogen bonds strengths in these systems were discussed based on cyclic hydrogen bond motifs (Pourayoubi *et al.*, 2011). It was concluded that the *R*_2_^2^(8) ring motif is generated by a pair of P(═O)···H–N_C(O)NHP(O)_ hydrogen bonds between two neighboring molecules in the crystal packing of phosphoric triamides of the formula *R*C(O)NHP(O)[N*R*^1^*R*^2^]_2_ which contain a *syn* orientation of P(═O) versus N—H. In the case of phosphoric triamides of the formula *R*C(O)NHP(O)[NH*R*^1^]_2_, crystal structure is usually composed of a chain of *R*_2_^2^(8) and *R*_2_^2^(12) ring motifs which alternately connected to each other. However, a few other hydrogen bond patterns were also found. The *R*_2_^2^(8) motif is formed by two P(═O)···H–N_C(O)NHP(O)_ hydrogen bonds and the *R*_2_^2^(12) motif by two C(═O)···H–N_amide_ hydrogen bonds. In this work, the synthesis and crystal structure of a new phosphoric triamide, P(O)[NHC(O)C_6_H_4_(3-F)][NH–C_6_H_4_–4–CH_3_]_2_, is reported. This investigation was carried out as part of a comprehensive study on the hydrogen bonds pattern in phosphoric triamides with formula *R*C(O)NHP(O)[NH*R*^1^]_2_. The phosphorus atom has a distorted tetrahedral environment (Fig. 1). Comparison of the O–P–N angles indicates that the O–P–N1 angle is smaller than ideal tetrahedral (107.02 (7)°) and the O–P–N2 and O–P–N3 angles (113.75 (7)° and 116.29 (7)°, respectively) display larger than ideal values. This probably arises due to steric repulsion involving the P(═O) group. Moreover, there is no π···π interaction between the two *para*-methyl phenyl groups. The C(═O) and P(═O) groups of the C(═O)NHP(═O) moiety are in *anti* positions relative to each other, contrary to the *syn* orientation of P(═O) and NH groups. The P(═O), C(═O) and P–N bond lengths and P–N–C bond angles are in the range of the expected values. In the crystal structure, molecules are linked through P(═O)···H–N_C(O)NHP(O)_ and C(═ O)···H–N_amide_ hydrogen bonds (Table 1), to give a linear chain running along the *b* axis.

### Experimental

Synthesis of 3-F–C_6_H_4_C(O)NHP(O)Cl_2_ A mixture of phosphorus pentachloride (3.773 g, 18.12 mmol) and 3-fluorobenzamide (2.521 g, 18.12 mmol) were refluxed in CCl_4_ for 8 h, and then the resulting solution was cooled to the room temperature. Formic acid (0.834 g, 18.12 mmol) was syringed dropwise into the stirring solution in 20 min and stirred for 6 h to yield the white precipitate that was filtered and dried in vacuum.

Synthesis of the title molecule To a solution of 3-F–C_6_H_4_C(O)NHP(O)Cl_2_ (0.256 g, 1 mmol) in CHCl_3_ (20 ml), a mixture of *p*-toluidine (0.214 g, 2 mmol) and triethylamine (0.202 g, 2 mmol) in CHCl_3_ (5 ml) was added dropwise at 273 K. After 4 h stirring, the solvent was evaporated in vacuum and then the resulting solid was washed with distilled water. Single crystals of title compound were obtained from a mixture of CH_3_OH, CH_3_CN and n-C_6_H_14_ after slow evaporation at room temperature. IR (KBr, cm^-1^): 3355 (NH), 3313 (NH), 3081 (NH), 2921, 1651 (C═O), 1615, 1588, 1513, 1440, 1386, 1267, 1235, 1210, 961, 870, 861, 817, 751.

### Refinement

All H atoms were placed in calculated positions with C—H = 0.93-0.96Å; N—H = 0.86Å and were included in a riding-model approximation with U_iso_(H) = 1.2U_eq_(C,N) or 1.5U_eq_(C_methyl_).

### Crystal data

**Table:**

C_21_H_21_FN_3_O_2_P	*F*(000) = 832
*M**_r_* = 397.38	*D*_x_ = 1.296 Mg m^−^^3^
Monoclinic, *P*2_1_/*n*	Mo *K*α radiation, λ = 0.71073 Å
Hall symbol: -P 2yn	Cell parameters from 6910 reflections
*a* = 10.2132 (5) Å	θ = 2.3–25.1°
*b* = 9.8588 (4) Å	µ = 0.17 mm^−^^1^
*c* = 20.2711 (9) Å	*T* = 296 K
β = 93.621 (2)°	Cubic, colorless
*V* = 2037.02 (16) Å^3^	0.25 × 0.22 × 0.14 mm
*Z* = 4	

### Data collection

**Table:**

Bruker APEXII CCD diffractometer	3844 independent reflections
Radiation source: fine-focus sealed tube	3061 reflections with *I* > 2σ(*I*)
graphite	*R*_int_ = 0.030
φ and ω scans	θ_max_ = 25.7°, θ_min_ = 2.9°
Absorption correction: multi-scan (*SADABS*; Bruker, 2007)	*h* = −12→12
*T*_min_ = 0.662, *T*_max_ = 0.745	*k* = −12→11
20105 measured reflections	*l* = −24→24

### Refinement

**Table:**

Refinement on *F*^2^	Primary atom site location: structure-invariant direct methods
Least-squares matrix: full	Secondary atom site location: difference Fourier map
*R*[*F*^2^ > 2σ(*F*^2^)] = 0.039	Hydrogen site location: inferred from neighbouring sites
*wR*(*F*^2^) = 0.109	H-atom parameters constrained
*S* = 1.04	*w* = 1/[σ^2^(*F*_o_^2^) + (0.0537*P*)^2^ + 0.6292*P*] where *P* = (*F*_o_^2^ + 2*F*_c_^2^)/3
3844 reflections	(Δ/σ)_max_ = 0.003
255 parameters	Δρ_max_ = 0.37 e Å^−^^3^
0 restraints	Δρ_min_ = −0.35 e Å^−^^3^

### Special details

**Table:**

Geometry. All s.u.'s (except the s.u. in the dihedral angle between two l.s. planes) are estimated using the full covariance matrix. The cell s.u.'s are taken into account individually in the estimation of s.u.'s in distances, angles and torsion angles; correlations between s.u.'s in cell parameters are only used when they are defined by crystal symmetry. An approximate (isotropic) treatment of cell s.u.'s is used for estimating s.u.'s involving l.s. planes.
Refinement. Refinement of *F*^2^ against ALL reflections. The weighted *R*-factor *wR* and goodness of fit *S* are based on *F*^2^, conventional *R*-factors *R* are based on *F*, with *F* set to zero for negative *F*^2^. The threshold expression of *F*^2^ > 2σ(*F*^2^) is used only for calculating *R*-factors(gt) *etc*. and is not relevant to the choice of reflections for refinement. *R*-factors based on *F*^2^ are statistically about twice as large as those based on *F*, and *R*- factors based on ALL data will be even larger.

### Fractional atomic coordinates and isotropic or equivalent isotropic displacement parameters (Å^2^)

**Table:**

	*x*	*y*	*z*	*U*_iso_*/*U*_eq_	
P1	0.55273 (4)	0.28199 (4)	0.97803 (2)	0.03501 (15)	
F1	0.00592 (13)	0.50210 (15)	1.10740 (8)	0.0863 (5)	
O1	0.32311 (13)	0.10156 (12)	0.97315 (7)	0.0522 (4)	
O2	0.62121 (12)	0.41249 (11)	0.97696 (6)	0.0424 (3)	
N1	0.39319 (14)	0.31600 (13)	0.98654 (7)	0.0384 (4)	
H1	0.3699	0.3994	0.9901	0.046*	
N2	0.56968 (16)	0.18952 (15)	0.91224 (7)	0.0457 (4)	
H2	0.5594	0.1032	0.9149	0.055*	
N3	0.59807 (15)	0.17934 (14)	1.03822 (7)	0.0407 (4)	
H3	0.6244	0.1001	1.0273	0.049*	
C1	0.0244 (2)	0.3949 (2)	1.06744 (11)	0.0523 (5)	
C2	0.14869 (18)	0.36985 (19)	1.04838 (10)	0.0462 (5)	
H2A	0.2185	0.4259	1.0621	0.055*	
C3	0.16722 (17)	0.25899 (17)	1.00822 (9)	0.0381 (4)	
C4	0.29931 (18)	0.21885 (16)	0.98807 (9)	0.0375 (4)	
C5	0.60112 (19)	0.24700 (18)	0.85011 (9)	0.0439 (4)	
C6	0.7180 (2)	0.3164 (2)	0.84531 (11)	0.0558 (5)	
H6	0.7763	0.3256	0.8822	0.067*	
C7	0.7477 (3)	0.3719 (3)	0.78558 (12)	0.0684 (7)	
H7	0.8258	0.4196	0.7831	0.082*	
C8	0.6657 (3)	0.3589 (2)	0.72965 (11)	0.0726 (7)	
C9	0.7009 (4)	0.4217 (3)	0.66478 (14)	0.1173 (13)	
H9A	0.7807	0.3818	0.6512	0.176*	
H9B	0.7130	0.5176	0.6705	0.176*	
H9C	0.6314	0.4053	0.6316	0.176*	
C10	0.06158 (19)	0.1775 (2)	0.98825 (10)	0.0492 (5)	
H10	0.0741	0.1025	0.9616	0.059*	
C11	−0.0622 (2)	0.2074 (2)	1.00776 (12)	0.0601 (6)	
H11	−0.1329	0.1531	0.9936	0.072*	
C12	−0.0815 (2)	0.3170 (2)	1.04808 (12)	0.0591 (6)	
H12	−0.1645	0.3374	1.0617	0.071*	
C13	0.59854 (18)	0.20754 (18)	1.10666 (9)	0.0410 (4)	
C14	0.6514 (2)	0.1138 (2)	1.15103 (10)	0.0577 (5)	
H14	0.6853	0.0331	1.1357	0.069*	
C15	0.6545 (3)	0.1384 (3)	1.21817 (12)	0.0753 (7)	
H15	0.6903	0.0735	1.2473	0.090*	
C16	0.6059 (3)	0.2569 (3)	1.24288 (11)	0.0699 (7)	
C17	0.5550 (3)	0.3505 (3)	1.19855 (12)	0.0721 (7)	
H17	0.5225	0.4317	1.2142	0.087*	
C18	0.5505 (2)	0.3279 (2)	1.13088 (11)	0.0609 (6)	
H18	0.5154	0.3933	1.1019	0.073*	
C19	0.6086 (4)	0.2837 (4)	1.31697 (12)	0.1036 (11)	
H19A	0.6172	0.3794	1.3250	0.155*	
H19B	0.6818	0.2370	1.3386	0.155*	
H19C	0.5286	0.2517	1.3340	0.155*	
C20	0.5180 (2)	0.2333 (2)	0.79481 (11)	0.0633 (6)	
H20	0.4392	0.1869	0.7974	0.076*	
C21	0.5505 (3)	0.2879 (3)	0.73502 (11)	0.0772 (8)	
H21	0.4936	0.2766	0.6978	0.093*	

### Atomic displacement parameters (Å^2^)

**Table:**

	*U*^11^	*U*^22^	*U*^33^	*U*^12^	*U*^13^	*U*^23^
P1	0.0371 (3)	0.0248 (2)	0.0440 (3)	0.00215 (18)	0.00981 (19)	−0.00342 (18)
F1	0.0618 (9)	0.0742 (9)	0.1262 (13)	0.0042 (7)	0.0322 (8)	−0.0372 (9)
O1	0.0543 (8)	0.0277 (6)	0.0757 (9)	−0.0005 (6)	0.0121 (7)	−0.0088 (6)
O2	0.0414 (7)	0.0284 (6)	0.0589 (8)	−0.0007 (5)	0.0146 (6)	−0.0053 (6)
N1	0.0370 (8)	0.0245 (7)	0.0543 (9)	0.0032 (6)	0.0083 (7)	−0.0023 (6)
N2	0.0605 (10)	0.0281 (7)	0.0503 (9)	−0.0020 (7)	0.0171 (8)	−0.0054 (7)
N3	0.0478 (9)	0.0273 (7)	0.0477 (9)	0.0088 (6)	0.0078 (7)	−0.0044 (6)
C1	0.0467 (11)	0.0432 (11)	0.0681 (13)	0.0050 (9)	0.0131 (10)	−0.0026 (10)
C2	0.0386 (10)	0.0372 (10)	0.0634 (12)	−0.0022 (8)	0.0082 (9)	−0.0043 (9)
C3	0.0394 (10)	0.0312 (8)	0.0441 (10)	−0.0004 (7)	0.0050 (8)	0.0047 (7)
C4	0.0427 (10)	0.0277 (8)	0.0424 (9)	−0.0009 (7)	0.0040 (8)	−0.0001 (7)
C5	0.0524 (11)	0.0362 (9)	0.0443 (10)	0.0017 (8)	0.0137 (9)	−0.0071 (8)
C6	0.0523 (12)	0.0636 (13)	0.0527 (12)	−0.0067 (10)	0.0117 (10)	−0.0048 (10)
C7	0.0773 (16)	0.0674 (15)	0.0634 (15)	−0.0214 (13)	0.0275 (13)	−0.0077 (12)
C8	0.113 (2)	0.0568 (13)	0.0499 (13)	−0.0161 (14)	0.0230 (14)	−0.0076 (11)
C9	0.200 (4)	0.098 (2)	0.0569 (16)	−0.045 (3)	0.036 (2)	0.0000 (16)
C10	0.0486 (12)	0.0433 (10)	0.0556 (12)	−0.0079 (9)	0.0027 (9)	−0.0022 (9)
C11	0.0420 (12)	0.0631 (14)	0.0747 (15)	−0.0108 (10)	−0.0004 (10)	0.0009 (12)
C12	0.0368 (11)	0.0619 (13)	0.0796 (15)	0.0015 (10)	0.0108 (10)	0.0076 (12)
C13	0.0419 (10)	0.0363 (9)	0.0450 (10)	−0.0035 (8)	0.0052 (8)	−0.0018 (8)
C14	0.0731 (15)	0.0432 (11)	0.0556 (13)	0.0021 (10)	−0.0059 (11)	0.0006 (9)
C15	0.110 (2)	0.0598 (14)	0.0541 (14)	−0.0110 (14)	−0.0124 (13)	0.0081 (12)
C16	0.0890 (18)	0.0740 (16)	0.0471 (12)	−0.0304 (14)	0.0079 (12)	−0.0068 (12)
C17	0.0956 (19)	0.0633 (14)	0.0590 (14)	−0.0005 (14)	0.0174 (13)	−0.0187 (12)
C18	0.0824 (16)	0.0496 (12)	0.0512 (12)	0.0142 (11)	0.0073 (11)	−0.0072 (10)
C19	0.148 (3)	0.116 (2)	0.0475 (14)	−0.048 (2)	0.0094 (16)	−0.0108 (15)
C20	0.0697 (15)	0.0617 (14)	0.0588 (13)	−0.0191 (12)	0.0068 (11)	−0.0095 (11)
C21	0.107 (2)	0.0759 (17)	0.0473 (13)	−0.0201 (16)	−0.0021 (13)	−0.0073 (12)

### Geometric parameters (Å, °)

**Table:**

P1—O2	1.4652 (12)	C9—H9A	0.9600
P1—N3	1.6297 (15)	C9—H9B	0.9600
P1—N2	1.6336 (15)	C9—H9C	0.9600
P1—N1	1.6830 (14)	C10—C11	1.380 (3)
F1—C1	1.352 (2)	C10—H10	0.9300
O1—C4	1.224 (2)	C11—C12	1.377 (3)
N1—C4	1.357 (2)	C11—H11	0.9300
N1—H1	0.8600	C12—H12	0.9300
N2—C5	1.436 (2)	C13—C14	1.376 (3)
N2—H2	0.8600	C13—C18	1.386 (3)
N3—C13	1.415 (2)	C14—C15	1.381 (3)
N3—H3	0.8600	C14—H14	0.9300
C1—C12	1.364 (3)	C15—C16	1.376 (4)
C1—C2	1.373 (3)	C15—H15	0.9300
C2—C3	1.383 (3)	C16—C17	1.367 (4)
C2—H2A	0.9300	C16—C19	1.523 (3)
C3—C10	1.385 (3)	C17—C18	1.388 (3)
C3—C4	1.488 (2)	C17—H17	0.9300
C5—C20	1.369 (3)	C18—H18	0.9300
C5—C6	1.385 (3)	C19—H19A	0.9600
C6—C7	1.380 (3)	C19—H19B	0.9600
C6—H6	0.9300	C19—H19C	0.9600
C7—C8	1.373 (4)	C20—C21	1.386 (3)
C7—H7	0.9300	C20—H20	0.9300
C8—C21	1.378 (4)	C21—H21	0.9300
C8—C9	1.517 (3)		
			
O2—P1—N3	116.29 (8)	C8—C9—H9C	109.5
O2—P1—N2	113.75 (7)	H9A—C9—H9C	109.5
N3—P1—N2	103.00 (8)	H9B—C9—H9C	109.5
O2—P1—N1	107.02 (7)	C11—C10—C3	120.22 (19)
N3—P1—N1	106.15 (7)	C11—C10—H10	119.9
N2—P1—N1	110.37 (8)	C3—C10—H10	119.9
C4—N1—P1	123.49 (12)	C12—C11—C10	120.4 (2)
C4—N1—H1	118.3	C12—C11—H11	119.8
P1—N1—H1	118.3	C10—C11—H11	119.8
C5—N2—P1	122.47 (12)	C1—C12—C11	118.13 (19)
C5—N2—H2	118.8	C1—C12—H12	120.9
P1—N2—H2	118.8	C11—C12—H12	120.9
C13—N3—P1	126.55 (12)	C14—C13—C18	118.45 (19)
C13—N3—H3	116.7	C14—C13—N3	119.09 (17)
P1—N3—H3	116.7	C18—C13—N3	122.44 (17)
F1—C1—C12	118.31 (18)	C13—C14—C15	120.7 (2)
F1—C1—C2	118.40 (19)	C13—C14—H14	119.7
C12—C1—C2	123.3 (2)	C15—C14—H14	119.7
C1—C2—C3	118.11 (18)	C16—C15—C14	121.4 (2)
C1—C2—H2A	120.9	C16—C15—H15	119.3
C3—C2—H2A	120.9	C14—C15—H15	119.3
C2—C3—C10	119.83 (17)	C17—C16—C15	117.7 (2)
C2—C3—C4	122.14 (16)	C17—C16—C19	120.9 (3)
C10—C3—C4	117.95 (16)	C15—C16—C19	121.4 (3)
O1—C4—N1	120.64 (16)	C16—C17—C18	121.9 (2)
O1—C4—C3	121.13 (16)	C16—C17—H17	119.0
N1—C4—C3	118.22 (14)	C18—C17—H17	119.0
C20—C5—C6	118.93 (19)	C13—C18—C17	119.8 (2)
C20—C5—N2	121.18 (18)	C13—C18—H18	120.1
C6—C5—N2	119.89 (18)	C17—C18—H18	120.1
C7—C6—C5	119.7 (2)	C16—C19—H19A	109.5
C7—C6—H6	120.1	C16—C19—H19B	109.5
C5—C6—H6	120.1	H19A—C19—H19B	109.5
C8—C7—C6	122.1 (2)	C16—C19—H19C	109.5
C8—C7—H7	119.0	H19A—C19—H19C	109.5
C6—C7—H7	119.0	H19B—C19—H19C	109.5
C7—C8—C21	117.5 (2)	C5—C20—C21	120.5 (2)
C7—C8—C9	120.8 (3)	C5—C20—H20	119.8
C21—C8—C9	121.7 (3)	C21—C20—H20	119.8
C8—C9—H9A	109.5	C8—C21—C20	121.3 (2)
C8—C9—H9B	109.5	C8—C21—H21	119.4
H9A—C9—H9B	109.5	C20—C21—H21	119.4

### Hydrogen-bond geometry (Å, °)

**Table:**

*D*—H···*A*	*D*—H	H···*A*	*D*···*A*	*D*—H···*A*
N1—H1···O2^i^	0.86	1.97	2.7835 (18)	157.
N3—H3···O1^ii^	0.86	2.06	2.8972 (18)	165.

Symmetry codes: (i) −*x*+1, −*y*+1, −*z*+2; (ii) −*x*+1, −*y*, −*z*+2.
